# Rural Caregiving After Stroke: A Global Qualitative Evidence Synthesis

**DOI:** 10.1093/geroni/igaf122.2770

**Published:** 2025-12-31

**Authors:** Adrianne Smiley, Rita Jablonski, David Vance, Elizabeth Byrd

**Affiliations:** University of Pittsburgh, Pittsburgh, Pennsylvania, United States; University of Alabama at Birmingham, Birmingham, Alabama, United States; University of Alabama at Birmingham, Birmingham, Alabama, United States; The University of Alabama at Birmingham, Birmingham, Alabama, United States

## Abstract

Caregivers of stroke survivors in rural communities face geographic isolation with limited access to medical providers, which limits caregiver support and increases caregiver burden. A consolidated synthesis of the domestic and international narrative experiences of rural stroke survivor caregivers would inform interventions. We conducted a systematic search using the PRISMA 2020 framework and guidelines proposed by Bettany-Saltikov and McSherry (2016), focusing on qualitative studies published in PubMed, Embase, and PsycINFO biomedical research databases with no restrictions on location nor publication year. Two independent reviewers assessed studies for original research exploring narrative themes on caregiver burden among rural caregivers, 18 years and older, caring for stroke survivors. Eligible studies followed our indexed search strategy developed in collaboration with a health sciences librarian. Extracted data items included study identifiers, characteristics and main findings (narrative themes). A total of 13 studies representing 161 caregivers, between the ages of 21-85 years old, were eligible for inclusion in this evidence synthesis. Descriptive statistics were used to summarize data items; stratification was applied to organize data into meaningful subgroups. Thematic content analysis and open coding were used to identify patterns and themes. Rural caregivers’ burden was alleviated by community and emotional support from their social networks and care providers but exacerbated by stroke survivor disability severity, unanticipated and rapid role transitions, delays in service initiation, inadequate communication from providers and mistrust in their local health system. Future interventions informed by these data may improve stroke survivors’ quality of life by reducing caregiver burden.

